# SARM1 Depletion Slows Axon Degeneration in a CNS Model of Neurotropic Viral Infection

**DOI:** 10.3389/fnmol.2022.860410

**Published:** 2022-04-13

**Authors:** Colin L. Crawford, Christina Antoniou, Lina Komarek, Verena Schultz, Claire L. Donald, Paul Montague, Susan C. Barnett, Christopher Linington, Hugh J. Willison, Alain Kohl, Michael P. Coleman, Julia M. Edgar

**Affiliations:** ^1^Institute of Infection, Immunity and Inflammation, College of Medical, Veterinary and Life Sciences, University of Glasgow, Glasgow, United Kingdom; ^2^John van Geest Centre for Brain Repair, Cambridge, United Kingdom; ^3^Department of Neurogenetics, Max Planck Institute of Experimental Medicine, Göttingen, Germany; ^4^MRC-University of Glasgow Centre for Virus Research, Glasgow, United Kingdom

**Keywords:** Zika virus, neurofilament, tubulin (microtubules), glia, nicotinamide adenine dinucleotide

## Abstract

Zika virus (ZIKV) is a neurotropic flavivirus recently linked to congenital ZIKV syndrome in children and encephalitis and Guillain-Barré syndrome in adults. Neurotropic viruses often use axons to traffic to neuronal or glial cell somas where they either remain latent or replicate and proceed to infect new cells. Consequently, it has been suggested that axon degeneration could represent an evolutionarily conserved mechanism to limit viral spread. Whilst it is not known if ZIKV transits in axons, we previously reported that ZIKV infection of glial cells in a murine spinal cord-derived cell culture model of the CNS is associated with a profound loss of neuronal cell processes. This, despite that postmitotic neurons are relatively refractory to infection and death. Here, we tested the hypothesis that ZIKV-associated degeneration of neuronal processes is dependent on activation of Sterile alpha and armadillo motif-containing protein 1 (SARM1), an NADase that acts as a central executioner in a conserved axon degeneration pathway. To test this, we infected wild type and *Sarm1* homozygous or heterozygous null cell cultures with ZIKV and examined NAD^+^ levels as well as the survival of neurons and their processes. Unexpectedly, ZIKV infection led to a rapid SARM1-independent reduction in NAD^+^. Nonetheless, the subsequent profound loss of neuronal cell processes was SARM1-dependent and was preceded by early changes in the appearance of β-tubulin III staining. Together, these data identify a role for SARM1 in the pathogenesis of ZIKV infection, which may reflect SARM1's conserved prodegenerative function, independent of its NADase activity.

## Introduction

The emergence of mosquito-borne Zika virus (ZIKV; a positive-strand RNA virus of the *Flaviviridae* family) in Pacific Ocean islands and the Americas added this previously little-known pathogen to the list of neurotropic flaviviruses (Baud et al., 2017; Gubler et al., 2017; Pierson and Diamond, 2018; Pardy and Richer, 2019). The association of ZIKV with multiple neurological complications including congenital ZIKV syndrome, encephalitis and Guillain-Barré syndrome (Gulland, 2016) makes it particularly important to understand the underlying mechanism. We previously demonstrated that post-mitotic murine CNS neurons are relatively refractory to infection by ZIKV, which productively infects oligodendroglia and astroglia in murine models of the perinatal CNS (Cumberworth et al., 2017; Schultz et al., 2021). Surprisingly, despite the paucity of infected neuronal somas and insignificant death of neurons, ZIKV infection led to a profound loss of axons and dendrites (Cumberworth et al., 2017). Thus, we hypothesized this involves a mechanism related to Wallerian degeneration, where axons also die independently from the soma (Coleman and Höke, 2020), following insults that include physical injury, toxins, and genetic mutation (Osterloh et al., 2012; Conforti et al., 2014; Coleman and Höke, 2020).

SARM1 (Sterile alpha and armadillo motif-containing protein 1) has a well-established role in Wallerian degeneration and is regarded as a central executioner in this conserved axon degeneration pathway involving NAD-related metabolism. The SARM1 TIR domain has NAD^+^ consuming glycohydrolase (NADase) activity, considered critical for its prodegenerative capacity (Essuman et al., 2017; Horsefield et al., 2019), although it has additional NADP hydrolase and base exchange activities that could also play important roles (Angeletti et al., 2021). As axons are often used by neurotropic viruses to enter neuronal and glial somas where the cellular machinery they use to replicate is located (Richards et al., 2021) it has been suggested that the known response of SARM1 to bunyavirus or rabies virus infection (both negative strand RNA viruses) could represent a mechanism to limit the spread of neurotropic virus (Mukherjee et al., 2013; Sundaramoorthy et al., 2020).

Notably, SARM1 also has a role in innate immunity since it contains a highly conserved Toll/Il-1 receptor (TIR) domain-containing protein, classified as a member of the toll-like receptor (TLR) adaptor family (Carty and Bowie, 2019). However, unlike other TLR adapters, SARM1 is predominantly expressed in neurons (Lin et al., 2014) and was initially described as an inhibitor of TLR signaling (Carty et al., 2006). Subsequent studies have suggested positive contributions of SARM1 to innate immunity through activation of transcriptional programmes that regulate the production of cytokines and chemokines, including in the nervous system (Carty and Bowie, 2019; Hopkins et al., 2021).

The SARM1-dependent loss of axons and dendrites with absent or delayed neuronal cell death following infection by bunyavirus (Mukherjee et al., 2013) or rabies virus (Sundaramoorthy et al., 2020) could thus provide an intriguing link between innate immunity and axon degeneration mechanisms. To investigate any role SARM1 may play in ZIKV-induced death of neuronal processes, we infected CNS myelinating spinal cord cultures from wild type mice, or *Sarm1* heterozygous or homozygous null mice (Kim et al., 2007) on a type I interferon receptor (*Ifnar1*) null background; the last to facilitate infection (Miner and Diamond, 2017). As previously (Cumberworth et al., 2017), *Sarm1* wild type neuronal soma were relatively refractory to infection, but glial cell infection was accompanied by progressive degeneration of neuronal processes. In contrast, there was prolonged survival of neuronal processes in *Sarm1* null cultures, accompanied by increased infection and death of neuronal somas. Surprisingly, this pro-degenerative role of SARM1 appeared to be independent of its NADase activity, although ZIKV infection itself caused NAD^+^ depletion in all three *Sarm1* genotypes.

## Methods and Materials

### Mice

Type I interferon receptor deficient (*Ifnar*1^−/−^) mice, which recapitulate aspects of human ZIKV infection and disease (Miner and Diamond, 2017) (129S7/SvEvBrdBkl-Hprtb-m2 background; B&K Universal) and *Sarm*1^−/−^ mice (maintained on a C57BL6/J background) (Kim et al., 2007) were bred to produce *Ifnar*1^+/−^::*Sarm*1^+/−^ mice. These were further crossed to produce *Ifnar*1^−/−^::*Sarm*1^+/−^ mice from which *Ifnar*1^−/−^::*Sarm*1^+/+^, ^+/−^ or ^−/−^ embryos were generated. Mice were maintained in Tecniplast 1284L Blue line IVC cages, in a 12-h light/dark cycle and provided *ad libitum* with sterile food and water. Mice were time-mated and pregnant females were killed by rising CO_2_ overdose and cervical dislocation on embryonic day (E) 13. All animal studies were approved by the Ethical Committee of the University of Glasgow and licensed by the UK Home Office (Project License number PPL P78DD6240).

### Myelinating Cell Cultures

Myelinating cell cultures were established using E13 mouse spinal cord as described in detail previously (Thomson et al., 2006; Bijland et al., 2019), with the exception that cultures were generated from single embryos rather than pooled embryos. A small piece of tail (~1 mm) was taken for retrospective genotyping.

### Genotyping

Genomic DNA was extracted from ear (adult) or tail (embryo) biopsies using a protocol modified from Truett et al. (2000). Briefly, tissue was lysed at 95°C for 90 min in 50 mM NaOH. Following neutralization with 10% v/v 1 M Tris pH 5, the resultant solution was vortexed and diluted 1:10 in nuclease-free water. *Ifnar1* genotyping was conducted as described previously (Cumberworth et al., 2017). For *Sarm1* genotyping, 1 μl of the diluted genomic DNA lysate was amplified in a 12.5 μl PCR reaction in a 1x RedTaq Reaction mix (Sigma-Aldrich) comprising 0.4 mM dNTP, 0.06 U/μl RedTaq Polymerase, 3 mM MgCl_2_ and 0.2 μM *Sarm1* primers. Cycling parameters were an initial denaturation at 95°C for 5 min, then 35 cycles (95°C 1 min; 60°C 1 min; 72°C 1 min) followed by a final elongation at 72°C for 10 min. The PCR products were resolved on standard 2% TAE agarose gels.

*Sarm1* primers were described previously (Gilley et al., 2015). Primer pairs 5′-ACGCCTGGTTTCTTACTCTACG-3′ and 5′- CCTTACCTCTTGC GGGTGATGC-3′ amplify wild type *Sarm1*, producing a 508-bp product. Primers 5′-GGTAGCCGGATCAAGCGTATGC-3′ and 5′- CTCATCTCCGGGCCTTTCGACC-3′ amplifies the neomycin resistance cassette retained in the knockout allele (Kim et al., 2007), producing a 450-bp product.

### Infection of Cultures With ZIKV

The Brazilian strain of ZIKV, ZIKV/H. sapiens/Brazil/PE243/2015 (GenBank accession number KX197192; abbreviated ZIKV PE243; referred to subsequently as ZIKV) has been described previously (Donald et al., 2016). CNS cultures were infected with ZIKV at a multiplicity of infection (MOI) of 0.3 or 0.6 for 1 h at 37°C in 2% fetal bovine serum (FBS) in cell culture grade PBS (10010023, Gibco™) as described previously (Cumberworth et al., 2017). Controls (mock-infected) were treated in parallel with vehicle only (2% FBS in PBS). Following incubation, the inoculum was aspirated, and the cultures returned to serum-free differentiation medium (Bijland et al., 2019). Media was partially replenished every 2 days.

For immunocytochemistry, cultures were fixed at 24 h post infection (hpi) or 6 days post infection (dpi) in 8% paraformaldehyde for 1 h at room temperature and subsequently stored in PBS at 4°C before staining. For NAD^+^ assay, cells were collected at 24 hpi.

### Measurements of NAD^+^ Levels

Samples were prepared for NAD^+^ quantification using the NAD/NADH-Glo™ assay (G9071, Promega) according to the manufacturer's instructions. Briefly, at 24 hpi, a cell scraper was used to remove cell monolayers from glass coverslips (2 × 35 mm dish per sample, 3 coverslips per dish) and these were transferred to a microcentrifuge tube. The cells were pelleted by centrifugation on a benchtop centrifuge at 13,000 RPM for 30 s. Supernatant was discarded and cells rinsed once in 1 ml PBS containing cOmplete™ EDTA-free protease inhibitor (11873580001, Sigma-Aldrich) before repeat centrifugation. The cells were lysed in 100 μl of bicarbonate-based lysis buffer (100 mM Na_2_CO_3_, 20 mM NaHCO_3_, 10 mM nicotinamide, 0.05% Triton-X 100, 1% dodecyltrimethylammonium bromide) by vortexing for ~1 min. Cell debris and insoluble material was then pelleted by centrifugation at 13,000 RPM for 10 min before the supernatant was transferred to a fresh 1.5 ml reaction tube. Pierce™ BCA Protein Assay Kit (23225, Thermo Fisher) was used according to the manufacturer's instructions to determine protein concentrations. Cell lysates were prepared to 0.5 mg/ml in lysis solution. 12.5 μg lysate was then incubated in 12.5 μl 0.4 M HCl at 60°C for 15 min, allowed to cool to room temperature, and neutralized with 12.5 μl 0.5 M Tris base. The samples were then snap-frozen and transported between institutions on dry ice. For determination of NAD^+^ levels, 10 μl of each neutralized reaction was mixed with 10 μl of the NAD-Glo detection reagent (prepared following manufacturer's instructions) on ice, in wells of a 384-well white polystyrene microplate (Corning). The plate and contents were incubated at room temperature for 50 min before luminescence was read using a GloMax^®^ Explorer plate reader (Promega). Concentrations of NAD^+^ are expressed as nmol/mg of protein.

### Immunocytochemistry

Post-fixation, cells were permeabilized in ethanol (−20°C; 10 min) and incubated in primary antibodies in blocking buffer (10% goat serum in PBS containing 2x NaCl [274 mM final] and 1% BSA) overnight at 4°C. Mouse anti-ZIKV (Aalto Bio, clone 0302156; 1 in 500) was used in combination with rabbit anti-NeuN (Sigma-Aldrich, ABN78; 1 in 500). Mouse IgG1 SMI31 anti-phosphorylated heavy and medium chain neurofilament (BioLegend, 801601; 1 in 1,500) and mouse IgG2a anti-β-Tubulin III (Sigma-Aldrich, T8578; 1 in 200) were applied simultaneously. After washing, secondary antibodies (goat anti-mouse IgG1 Alexa 488/568/647 and goat anti-rabbit IgG or goat anti-mouse IgG2a Alexa 568/647; 1 in 1,000; Invitrogen) were applied for 1 h at room temperature. Coverslips were mounted on glass slides in Mowiol^®^ 4-88 (Sigma-Aldrich) mounting medium with DAPI (1 μg/ml; D1306, Invitrogen).

### Image Capture

For quantification of cell density and neurofilament staining, fluorescence microscopy and image capture were performed using a Zeiss Axio Imager M2 fluorescent microscope with standard epifluorescence optics and Zen Blue software (Carl Zeiss AG, Germany). To avoid bias, fields of view (FoV) were selected in the blue (DAPI) channel and images were captured (10 images from across each coverslip) at x20 magnification in the red, far red, green and blue channels. Representative images for illustration were obtained using the same microscope and software or with a Zeiss LSM 880 Confocal Microscope using a Zeiss Plan-Apochromat 63 × /1.4 oil immersion objective and Zen Black software.

### Quantification of Cells

A rectangular area of interest (AoI) of 111,488 μm^2^ was placed on each image and immunostained cells or DAPI +ve nuclei, respectively, within and touching west and north borders were quantified. Only immunopositive cells with a DAPI +ve nucleus qualified. The average cell density per AOI was converted to cells/mm^2^ using the formula, cell density per AOI/area of AOI μm^2^ × 1,000,000. Pyknotic nuclei were distinguished from healthy nuclei on the basis of size and homogeneity and intensity of DAPI staining; pyknotic nuclei being condensed and intensely labeled (Cummings and Schnellmann, 2004). Experimenters were blinded to genotypes and three (of nine) sets of images were each quantified by two experimenters (average values were used) for quality control purposes.

### Quantification of Neurofilament Staining

Digital images of representative fields of view obtained from cell cultures immunostained with antibody SMI31 that recognizes phosphorylated heavy (H) and medium (M) chain neurofilament, were used to quantify the densities of neuronal processes. A Fiji (Schindelin et al., 2012) macro was recorded to analyse all images identically. To specifically select linear structures, a threshold between 12 and 30 (depending on staining) was used and debris was filtered out using the “analyze particles” function with the following parameters: size = 250.00-infinity, circularity = 0.00–0.30. The “histogram” function was then used to obtain the number of positive pixels. Macro to quantify axon densities in Fiji:

setOption(“ScaleConversions”, true);

run(“8-bit”);

setAutoThreshold(“Default dark”);

//run(“Threshold...”);

setThreshold(12, 255);

run(“Analyze Particles...”, “size=250.00-Infinity circularity=0.00-0.30 show=Masks display in_situ”);

getHistogram(values, counts, 2);

Array.print(counts);

### Statistical Analysis

Analyses were performed using GraphPad Prism 9 software (GraphPad Software Inc., San Diego, CA). Significance is indicated as <0.05 (^*^), <0.001 (^***^) and <0.0001 (^****^). A one-way ANOVA was used to compare cell densities or neurofilament stained area across three *Sarm1* genotypes, using independent cultures each generated from a single embryo from a minimum of 3–4 independent litters per analysis. A two-way ANOVA was used to compare NAD^+^ concentrations between mock-infected and infected cultures, across all three genotypes, using independent cultures each generated from a single embryo from 3 independent dams.

## Results

### SARM1 Depletion Preserves Axons Following ZIKV Infection

**Figure 1:**
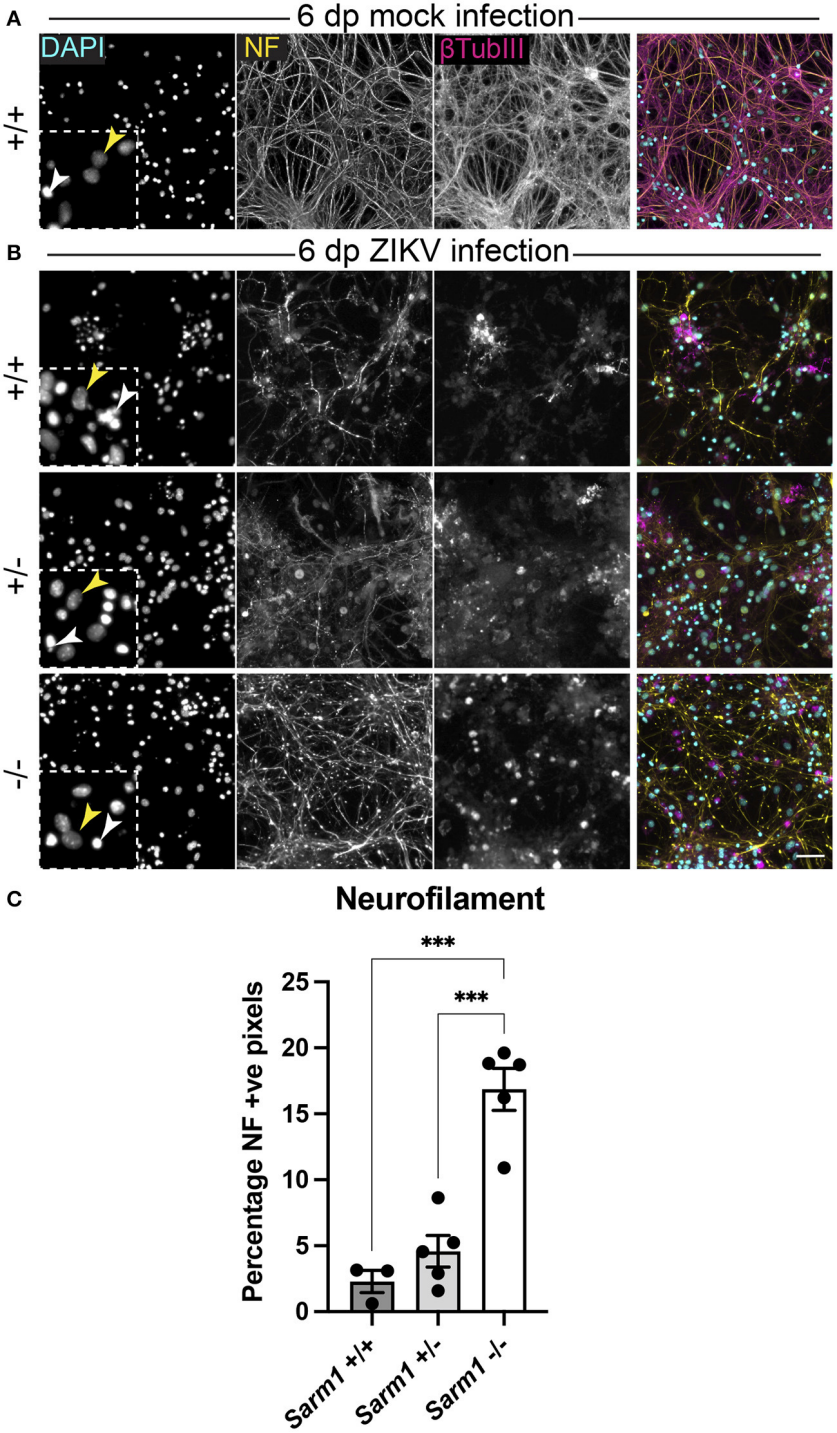
SARM1 depletion delays degeneration of neuronal processes. **(A)** Mock infected *Sarm*1^+/+^ cultures appeared healthy at 6 dpi, with numerous pale DAPI +ve nuclei (yellow arrowhead in inset), relatively few pyknotic nuclei (white arrowhead) and dense neurofilament (NF) and β-tubulin III (β-TubIII) positive cell processes. **(B)** ZIKV infected cultures appeared altered at the same time point, with several pyknotic nuclei (white arrowheads in insets) and fragmented neurofilament and β-tubulin III-stained neuronal processes. This was most obvious in *Sarm*1^+/+^ and *Sarm*1^+/−^ cultures whereas neurofilament appeared relatively preserved in *Sarm*1^−/−^ cultures. Bar: 50 μm. **(C)** Quantification of neurofilament positive pixels as a proportion of all pixels demonstrated a significant preservation of neuronal processes in *Sarm*1^−/−^ cultures compared to *Sarm*1^+/+^ or ^+/−^. Bars represent mean ± S.E.M. ***p < 0.001.

Embryonic murine spinal cord-derived myelinating cell cultures contain all major neural cell types including choline acetyltransferase positive motor neurons that project to the body's periphery *in vivo*, oligodendrocyte progenitors (OPCs), myelinating oligodendrocytes, astrocytes and microglia. To determine if SARM1 depletion (*Sarm*1^−/−^) or haploinsufficiency (*Sarm*1^+/−^) conserves axons, we infected cultures generated from *Sarm*1^+/+^ (control), *Sarm*1^+/−^ or *Sarm*1^−/−^ mice with MOI 0.3 or 0.6 ZIKV (Table 1) and stained them with antibody to phosphorylated H- and M-neurofilament or β-tubulin III, at 6 dpi (Figure 1). Quantification of neurofilament staining demonstrated statistically significant preservation of neuronal cell processes in infected *Sarm*1^−/−^ cultures compared to *Sarm*1^+/+^ controls. SARM1 haploinsufficiency conferred no benefit at this time point (Figure 1).

**Table 1:** MOI ZIKV used in experiments quantifying neurofilament and β-tubulin III staining.

	**WT**	**Het**	**KO**
MOI 0.3	1	3	3
MOI 0.6	2	2	2
*Total*	3	5	5

### Removing SARM1 Does Not Reduce Susceptibility of Cells to Infection

**Figure 2:**
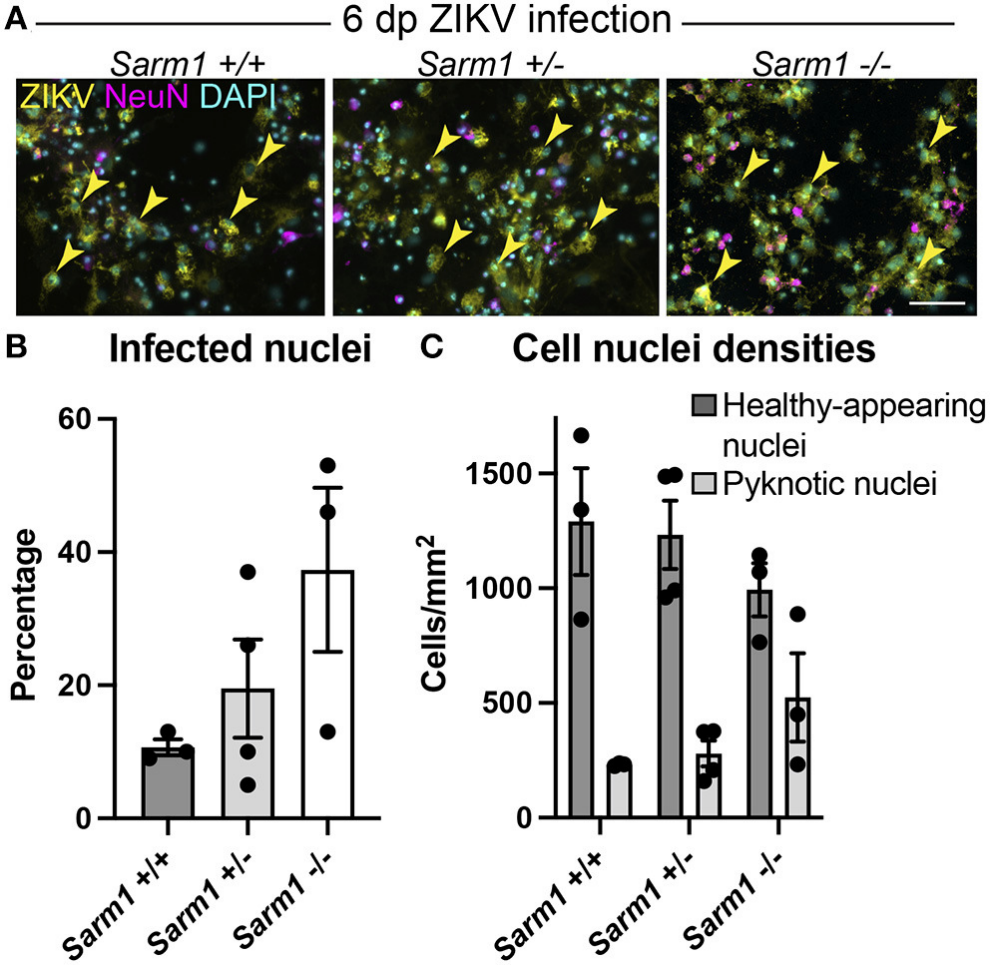
SARM1 does not enhance susceptibility to ZIKV infection. **(A)** Fluorescence micrographs of ZIKV infected cells at 6 dpi in *Sarm1* ^+/+^, ^+/−^ and ^−/−^ cultures. Across all three genotypes, many cells are ZIKV +ve (yellow arrowheads). Few of these are NeuN +ve, indicating that most infected cells are glia. Bar: 50 μm. **(B)** Graph of percentage DAPI +ve nuclei that are ZIKV +ve, demonstrates that SARM1 status does not result in significant difference in proportions of infected cells, although there is a trend toward an increased proportion of infected cells in the absence of SARM1. **(C)** Graphs of DAPI +ve cell densities demonstrates there is no significant difference in cell densities across the three *Sarm1* genotypes. However, there appears to be a tendency for increased cell death (pyknotic nuclei) in the absence of SARM1. Bars represent mean ± S.E.M.

To confirm that the observed decrease in degeneration of neuronal processes in the absence of SARM1 does not simply reflect reduced infection in *Sarm*1^−/−^ cultures, we quantified the proportion of DAPI +ve cell nuclei (both healthy-appearing and pyknotic) that were ZIKV +ve, in each of the three genotypes. We ruled out a decrease in the proportion of productively infected cells at 6 dpi, instead, observing a trend toward an increase in the proportion of infected cells in the *Sarm*1^−/−^ cultures (Figures 2A,B). We ruled out the possibility this was due to differences in cell densities between genotypes (Figures 2A,C). Thus, protection of neuronal processes does not result from there being reduced numbers of productively infected cells in the absence of SARM1.

### ZIKV Infection Leads to a Rapid Depletion of NAD^+^ Independent of *Sarm1* Genotype

**Figure 3:**
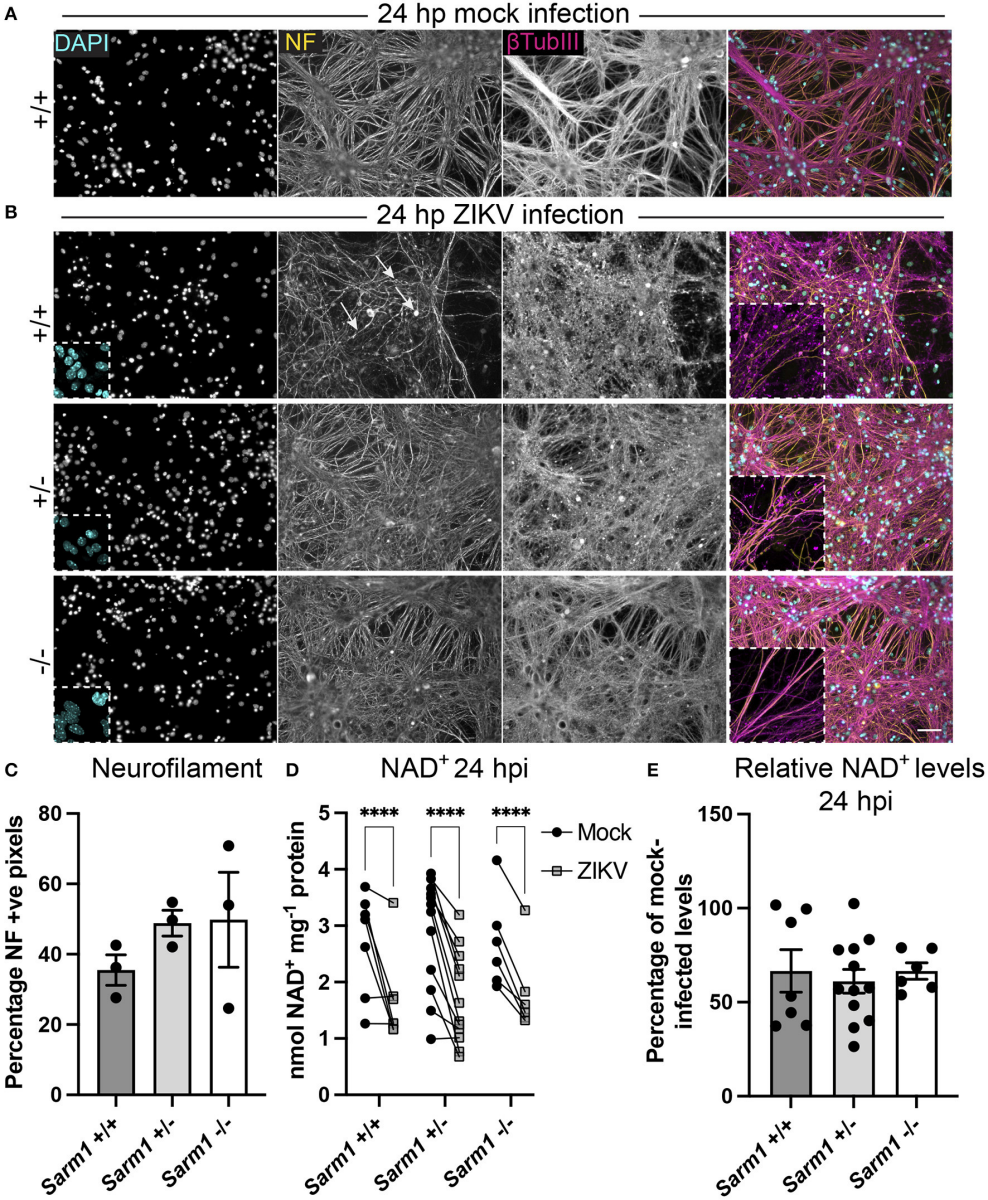
ZIKV infection depletes NAD^+^ levels independent of SARM1 at 24 hpi. **(A)** Mock infected *Sarm*1^+/+^ cultures appear healthy at 24 hpi. DAPI +ve cell nuclei are abundant and neurofilament (NF) and β-tubulin III-stained neuronal processes appear dense and smooth. Bar: 50 μm. **(B)** At 24 hpi with ZIKV, DAPI +ve cell nuclei appear similar in terms of density and appearance across all three *Sarm1* genotypes, although there is already evidence of subtle injury to neuronal cell processes in *Sarm*1^+/+^ (arrows indicate irregularly stained neurofilament +ve structures) and *Sarm*1^+/−^ cultures, most obvious with β-tubulin III staining. In contrast, neuronal processes in *Sarm*1^−/−^ cultures appear dense and smooth, as in the mock-infected controls (see also Supplementary Figure 1). **(C)** Neurofilament positive pixels as a percentage of all pixels per AOI is similar across all three genotypes suggesting that neuronal processes remain viable at this timepoint. **(D)** Compared to levels in matched mock-infected controls (black circles), NAD^+^ levels are significantly reduced in ZIKV-infected cultures (gray squares) at 24 hpi. **(E)** The relative reduction in NAD^+^ in ZIKV infected cultures in comparison to their mock-infected controls is similar across all three genotypes. Bars represent mean ± S.E.M. ****p < 0.0001.

SARM1 is an NADase that depletes axons of NAD^+^ following a variety of insults. Furthermore, the NS3 domain of ZIKV itself has been shown to deplete NAD^+^ by a PARP1-dependent mechanism in HeLa cells (Xu et al., 2019). To determine if there is depletion of NAD^+^ upon ZIKV infection of CNS cells, we first determined a time point to assay NAD^+^ concentrations, prior to overt cellular injury. At 24 hpi, staining of DAPI +ve cell nuclei revealed little difference between infected cultures of either of the three *Sarm1* genotypes and their mock-infected controls (Figures 3A,B and Supplementary Figure 1), suggesting the absence of overt ZIKV infection-related cell death at this time point, as quantified previously (Cumberworth et al., 2017). Nonetheless, despite that staining for phosphorylated H- and M-neurofilament (antibody clone SMI31) demonstrated similar positive pixel densities across all the three genotypes (Figure 3C), subtle changes in morphology of some neuronal processes in ZIKV-infected *Sarm*1^+/+^ cultures could be seen at this time point (arrows; Figure 3B). This was confirmed by β-tubulin III staining which provided evidence for fragmentation of the microtubule network in ZIKV-infected *Sarm*1^+/+^ and *Sarm*1^+/−^, but not *Sarm*1^−/−^ cultures or mock-infected controls (Figures 3A,B and Supplementary Figure 1). Quantification of NAD^+^ levels at 24 hpi demonstrated a similar significant decrease in NAD^+^ levels in ZIKV-infected cultures of each of the three *Sarm1* genotypes, in comparison to their matched mock-infected controls (Figure 3D). The degree of NAD^+^ reduction was most consistent in *Sarm*1^−/−^ cultures and more variable in wild type and heterozygous controls, most particularly when NAD^+^ levels were below 2 nmol/mg^−1^ protein in mock-infected control cultures. On average, NAD^+^ levels were reduced to ~60% of mock-infected control levels, across all three genotypes (Figure 3E). Together, these data demonstrate that whilst degeneration of neuronal processes is expediated by SARM1, NAD^+^ depletion at 24 hpi is independent of SARM1. NAD^+^ levels were not assessed at later time points due to confounding consequences of leakage of NAD^+^ from dying cells and injured axons.

### SARM1-Dependent Degeneration of Neuronal Cell Processes Preserves Neuronal Somas

**Figure 4:**
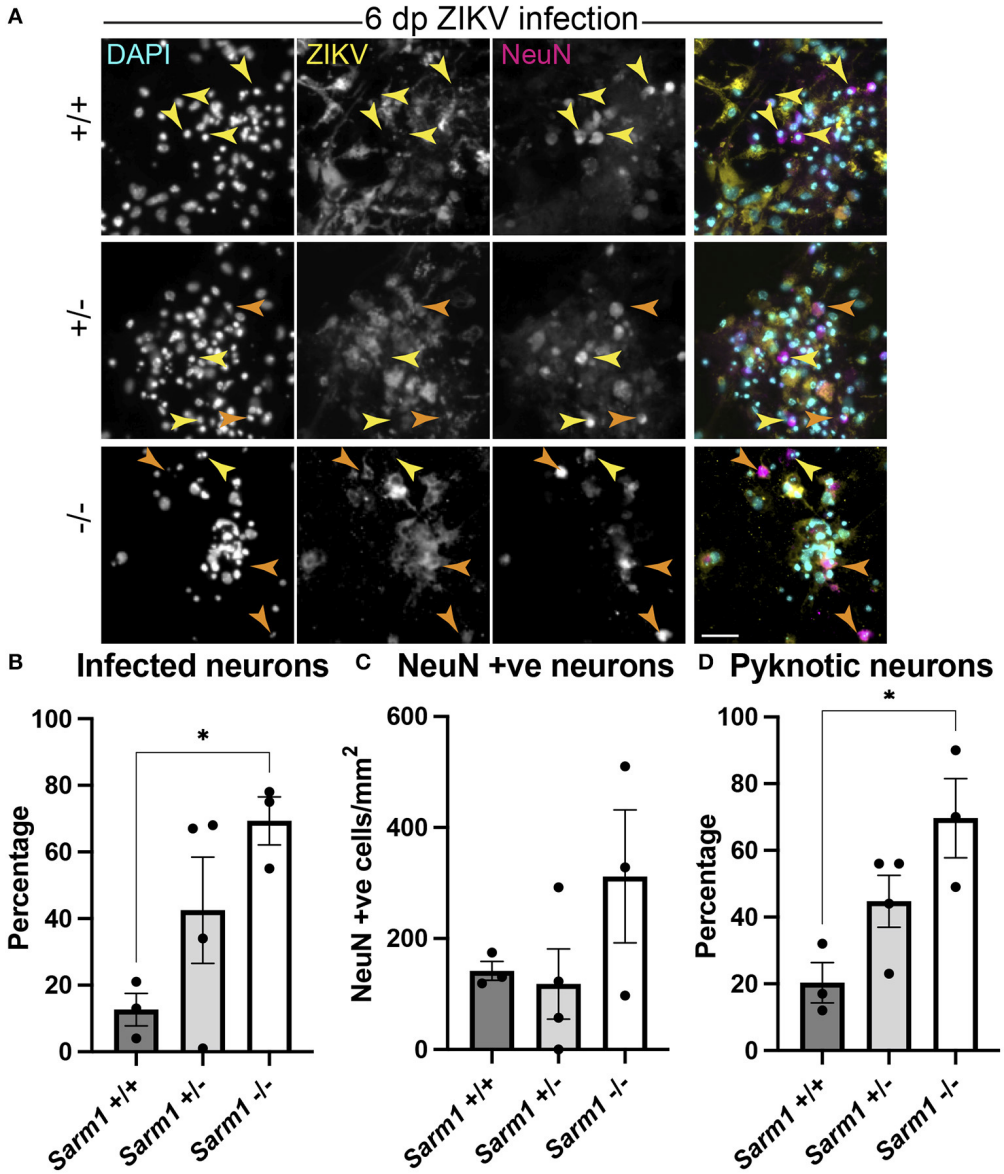
SARM1-dependent degeneration of neuronal processes limits infection and death of neurons. **(A)** Fluorescence micrographs of ZIKV infected *Sarm*1^+/+^, *Sarm*1^+/−^ and *Sarm*1^−/−^ cultures at 6 dpi. In wild type cultures (*Sarm*1^+/+^), most NeuN +ve neurons are not ZIKV +ve (yellow arrowheads) whereas in *Sarm*1^−/−^ cultures, many NeuN +ve neurons are ZIKV +ve (orange arrowheads). In *Sarm*1^+/−^ cultures, both ZIKV +ve (orange arrowheads) and -ve (yellow arrowheads) NeuN +ve cells are evident. Bar: 50 μm. **(B)** Graph of proportions of NeuN +ve infected cells, demonstrates a significantly greater proportion of NeuN +ve neurons are ZIKV +ve in *Sarm*1^−/−^ cultures compared to *Sarm*1^+/+^ control. **(C)** This does not reflect a significant difference in NeuN +ve cell densities in *Sarm*1^−/−^ cultures compared to *Sarm*1^+/+^ controls. **(D)** There is a significant increase in the proportion of pyknotic NeuN +ve nuclei in *Sarm*1^−/−^ cultures compared to *Sarm*1^+/+^ controls. Bars represent mean ± S.E.M. *p < 0.05 (defined in M&M).

As some neurotropic viruses use microtubule-dependent axon transport to reach the neuronal soma, the changes observed in microtubule staining at 24 hpi led us to ask whether SARM1-dependent mechanisms might limit infection of neuronal somas. To address this, we examined infected NeuN +ve somas across the three *Sarm1* genotypes (Figure 4A). Compared to *Sarm*1^+/+^, we observed a significant increase in the proportion of infected neuronal somas in *Sarm*1^−/−^ cultures and a similar trend in *Sarm*1^+/−^ cultures (Figure 4B), that did not reflect differences in the total number of NeuN positive cells of any genotype (Figure 4C). Correspondingly, we observed a significant increase in the proportion of pyknotic neurons in *Sarm*1^−/−^ cultures compared to *Sarm*1^+/+^ cultures. *Sarm*1^+/−^ cultures had, on average, an intermediate proportion of pyknotic neurons, likely reflecting SARM1 haploinsufficiency (Figure 4D). Together, these data suggest that SARM1-dependent degeneration of neuronal processes limits infection and death of neuronal somas.

## Discussion

Our data provide novel evidence of a SARM1-dependent mechanism of degeneration of neuronal processes following ZIKV infection. Further, this involves neither an increase in ZIKV infected cells nor increased susceptibility of neuronal somas to cell death. On the contrary, at least at the time point tested, the proportions of infected and/or dying neurons were significantly enhanced in the absence of SARM1. Furthermore, we provide evidence in primary CNS cell cultures for the physiological relevance of a previous report of ZIKV-induced NAD^+^ depletion in HeLa cells (Xu et al., 2019). Surprisingly, although NAD^+^ depletion has been suggested to make SARM1 activation more likely (Figley et al., 2021) we found no clear evidence of any additional depletion of NAD^+^ under conditions (ZIKV-infection) where SARM1 contributes to axon loss. Indeed, since most infected cells in our mixed neural cell cultures are glia (Cumberworth et al., 2017), it may be that depletion of glial NAD^+^ makes the largest contribution to the fall we observed. Irrespective of the cellular localization of NAD^+^ depletion, it appears that the mechanism by which SARM1 contributes to degeneration of neuronal processes following ZIKV infection is independent of its NADase activity.

One possible explanation for these findings is that degeneration of neuronal processes in this context could result from one of the additional enzyme activities of SARM1, which include NADP hydrolysis and cyclisation, and base exchange of nicotinamide of NAD(P) with other endogenous pyridine bases such as nicotinic acid (Zhao et al., 2019; Angeletti et al., 2021; Figley et al., 2021). These may lead to reduced ROS buffering capability due to lowering of NADPH, or to calcium mobilization due to generation of NaADP, the most potent known calcium mobilizing signal (Galione et al., 2000; Angeletti et al., 2021). Indeed, a *circa* 50% decline in NAD^+^, as observed here at 24 hpi, is not sufficient to kill axons or cells since doses of NAMPT inhibitor FK866 that depletes NAD^+^ to or beyond this level, are not lethal (Di Stefano et al., 2015). Transected *Sarm*1^−/−^ sciatic nerve axons also survive more than a week after the fall in NAD^+^ passes 50% of basal levels (Gilley et al., 2015). Thus, it will be important to investigate these other potential mediators of SARM1 activity, or indeed any novel activities that are found for this multifunctional enzyme, in future work.

One limitation of our study is that the NAD^+^ assay is based on cell culture lysates and therefore it cannot discriminate cell or cell compartment-specific effects. Regarding this, it is worth noting that ZIKV protein NS3 itself potently activates a nuclear poly (ADP-ribose) polymerase, PARP1, in HeLa and glioma cells that results in NAD^+^ depletion and cell death at 48 h post-treatment in the former (Xu et al., 2019). As PARP1 is also one of the main NAD^+^ consuming enzymes in neural cells (Klimova and Kristian, 2019), it seems reasonable to speculate that it is responsible for the *circa* 50% NAD^+^ depletion at 24 hpi in our ZIKV infected neural cell cultures. Consequently, we cannot exclude the possibility that a SARM1-dependent depletion of NAD^+^ specifically in neuronal processes is masked in these mixed neural cell type cultures. We also cannot rule out a SARM1-dependent fall in NAD^+^ at later timepoints, rapidly followed by degeneration of neuronal processes, since if this were to happen it would be confounded by cell death and extremely difficult to capture the optimal time point to detect it.

Degeneration of neuronal processes following ZIKV infection occurs independently of overt infection or death of neuronal somas in *Sarm1* wild type cultures [the current study and Cumberworth et al. (2017)]. This raises the intriguing possibility that it represents a neuroprotective mechanism designed to limit viral spread, as proposed previously (Mukherjee et al., 2013; Sundaramoorthy et al., 2020). Neuronal processes, especially axons, are highly reliant on microtubule-dependent transport for function and survival (De Vos et al., 2008; Magiera et al., 2018; Guedes-Dias and Holzbaur, 2019; Kelliher et al., 2019; Koppers and Farías, 2021), and several neurotropic viruses including Theiler's virus, herpes simplex virus, poliovirus, rabies virus, and West Nile virus (WNV), transit in axons, likely relying on microtubule-dependent transport to reach their targets (Salinas et al., 2009 and reviewed in Richards et al., 2021). Thus, the significant increase in infection and death of neuronal somas in SARM1-depleted cultures (with delayed degeneration of neuronal processes) suggests that ZIKV can be transported in this way too and that SARM1-dependent degeneration of neuronal processes limits its spread to neuronal somas. Nonetheless, the possibility that the SARM1-dependent intrinsic immune capacity of neurons (Wang et al., 2018) protects these cells in *Sarm*1^+/+^ cultures, should not be discounted.

It is noteworthy that widespread changes in neuronal-specific β-tubulin III immunostaining were observed as early as 24 h post-ZIKV infection. This raises the intriguing possibility that, more generally, activation of evolutionarily conserved SARM1 hampers the retrograde transportation of some viruses from the site of entry at the body's periphery, even prior to axon destruction. In support of this suggestion is the fact that SARM1 participates in microtubule posttranslational modification (Chen et al., 2011), suggesting that activation of SARM1 could have a rapid impact on motor protein binding and cargo (including virus) transport.

The initial trigger for SARM1-dependent degeneration of neuronal processes following ZIKV infection could act through gain or loss of function, for example, through cytotoxicity, failure of trophic support from neighboring glia, or a combination of both. If a soluble cytotoxic factor is involved, neuronal processes seem particularly susceptible since all cell types are, in principle, equally exposed in cell culture where they are bathed in media. Nitric oxide is one potential cytotoxic molecule that causes Wallerian degeneration changes in electrically active axons at low micromolar concentrations (Smith et al., 2001). Failure of support from oligodendrocytes and astrocytes (Stassart et al., 2018; Duncan et al., 2021; Veloz Castillo et al., 2021) infected with ZIKV is another possibility. These cells provide energy metabolites to neurons (Pellerin et al., 1998; Fünfschilling et al., 2012; Lee et al., 2012), although energy insufficiency due to failed energy substrate provision is unlikely in this context because neuronal processes are suffused in a glucose-rich media *in vitro*. Nonetheless, oligodendrocytes and astrocytes confer axon support through multiple mechanisms that could be relevant in the context of ZIKV infection, such as regulating ion and glutamate homeostasis (Verkhratsky and Nedergaard, 2018; Hart and Karimi-Abdolrezaee, 2021), performing anti-oxidant functions (Baxter and Hardingham, 2016; Mukherjee et al., 2020), and enhancing axonal energy metabolism by deacetylating mitochondrial proteins (Chamberlain et al., 2021).

CNS complications of ZIKV infection in adults are rare, but include encephalitis, meningitis, myelitis, meningoencephalitis, transverse myelitis and neuropsychiatric symptoms (Carteaux et al., 2016; Galliez et al., 2016; Mécharles et al., 2016; da Silva et al., 2017; Zucker et al., 2017; Brito Ferreira et al., 2020). However, ZIKV infection has been linked to the peripheral neuropathy, Guillain-Barré syndrome (Oehler et al., 2014; Pinto-Díaz et al., 2017). Roles for SARM1 in other viral neuropathies have been reported. Mice lacking SARM1 are more susceptible to WNV infection, with reduced survival and enhanced neuronal death in the brainstem (Szretter et al., 2009), and this has been confirmed in a new CRISPR/Cas9-generated SARM1 null mouse that excludes any contribution from passenger mutations co-inherited with SARM1 from the original 129 strain ES cells (Uccellini et al., 2020). Similarly, a recent study demonstrated that different field strains of lyssavirus (“rabies”) cause SARM1-dependent axon degeneration in CNS and PNS mouse neurons, further supporting a role of SARM1 in neurotropic viral infection (Sundaramoorthy et al., 2020). The authors showed that the SARM1-mediated axon loss prevents the spread of virus along interconnected neurons, suggesting a possible host-defense mechanism restricting virus infection. The subsequent loss of neuronal processes in this and our study, possibly as a result of such a neuronal defense response, would be consistent with our observation that ZIKV-induced neuronal soma death increases in the absence of SARM1, and could thus represent a mechanism underlying neurological complications in rabies and ZIKV infection. It may even help to explain the evolutionary advantage of a mechanism for rapid axon degeneration, which otherwise appears paradoxical. It will be of great interest to further understand the effects of these and other viruses on SARM1-dependent axon loss, on NAD^+^ levels, and the roles of these mechanisms in viral neuropathies.

## Data Availability Statement

The original contributions presented in the study are included in the article/Supplementary Materials, further inquiries can be directed to the corresponding author.

## Ethics Statement

The animal study was reviewed and approved by Ethics Review Committee of the University of Glasgow.

## Author Contributions

JE and MC: study design and supervision. CC, CA, LK, and VS: experimentation. CD: viral propagation. PM: genotyping. CC, LK, and JE: data analysis. JE, MC, CA, and CC: manuscript draft. JE, CC, and LK: figure composition. All authors: manuscript editing and approval. HW, JE, AK, MC, SB, and CL: funding. All authors contributed to the article and approved the submitted version.

## Funding

This project was partially funded through the European Union's Horizon 2020 research and innovation programme under ZikaPLAN Grant Agreement No. 734584 (HW, JE, SB, and CL); the UK Medical Research Council (MC_UU_12014/8 and MR/N017552/1) (AK); CSO and University of Glasgow SPRINT PhD programme studentship to CLC (JE); an MRC DTP Award and Gates Scholarship (CA); the John and Lucille van Geest Foundation (MC).

## Conflict of Interest

The authors declare that the research was conducted in the absence of any commercial or financial relationships that could be construed as a potential conflict of interest.

## Publisher's Note

All claims expressed in this article are solely those of the authors and do not necessarily represent those of their affiliated organizations, or those of the publisher, the editors and the reviewers. Any product that may be evaluated in this article, or claim that may be made by its manufacturer, is not guaranteed or endorsed by the publisher.
