# Supplementary figures and images for: SARM1 Depletion Slows Axon Degeneration in a CNS Model of Neurotropic Viral Infection
Source: Front Mol Neurosci. 2022 Apr 13;15:860410. doi: 10.3389/fnmol.2022.860410 (PMC9043327; doi:10.3389/fnmol.2022.860410)

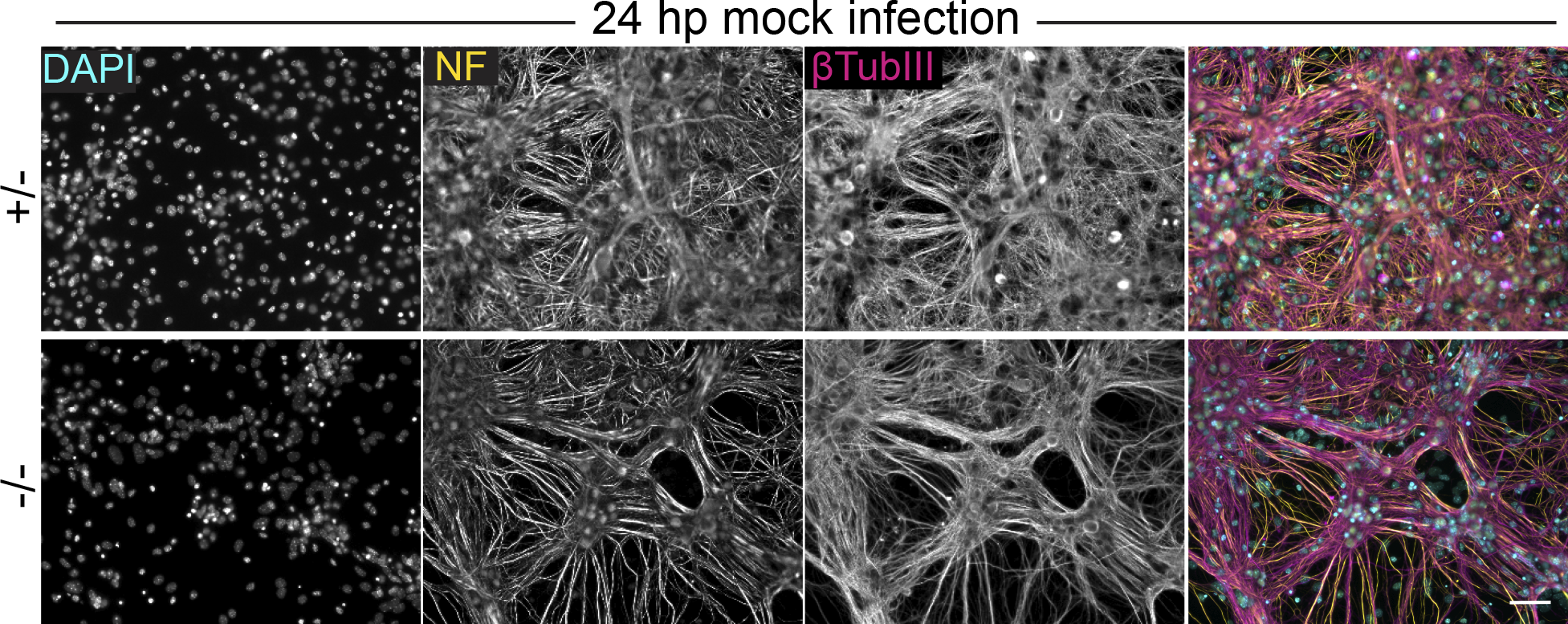

Supplement: Supplementary file 1 [file Image_1.TIF]
